# Vaccination in the Light of the Royal British Commission

**Published:** 1897-07

**Authors:** M. R. Leverson

**Affiliations:** Fort Hamilton, L. I., N. Y.


### VACCINATION IN THE LIGHT OF THE ROYAL BRITISH COMMISSION.

M. R. Leverson, M. D., Fort Hamilton, L. I., N. Y.

(Continued from page 225.)

In The Homeopathic Physician for April (page 119) was published the letter of the French Minister of War, saying that no records had been kept of the number of French soldiers who died of small-pox during the war of 1870-71, and on page 120 the statement was made that the same was the case with regard to the German army, but up to the time of going to press I had not been able to get a copy of the German letter.

Through the kindness and labors of Dr. Eugene Bettes, of Washington, D. C., a translation of that letter has been obtained in the following words:

No. 691-7 MMA.

"Army Medical Department, War Office, Berlin, July 30th, 1883.

"In reply to your letter of July 6th to His Excellency the Minister of War the Chief Clerk forwards a certified extract from the registrar of those who died of small-pox in the Prussian army in the several months of the years 1869, 1870, and 1871.

"For the time from July, 1870, to June, 1871 (the twelve months of the war), the number wished for are not recorded, and regret is expressed that on this account the desired information cannot be given. Toler Lisouke."

"To George S. Gibbs, Esq., Henry Lodge, Darlington, England."

The letter of retraction of Dr. Carpenter of the error (afterward rehashed by Dr. Hopkirk before the R. B. Commission) was published in the London *Daily News* August 7th, 1888.

How hard it is to kill a lie! Especially a good bouncing one, such as health (?) officers delight in!

We will now resume our abstract of the testimony:

The next witness was Francis Xavier Frederick MacCabe, Fellow of Kings and Queens College of Physicians of Ireland, and M. R. C. S. of England; Medical Commissioner on the Local Government Board for Ireland; prior to holding which office he had been one of the Board's Inspectors (Q. 3033-6). With reference to small-pox before the time of inoculation he quoted (Q. 3,038) from the fifth part of the Irish census, 1851, a record of small-pox in A. D. 675: "There reigned a kind of great leprosie in Ireland this year called the pox, in Irish *bolgach*," and said "various references from the year 675 downwards are to be found through these records to the prevalence of small-pox in Ireland." He refers to *The Annals of the Kingdom of Ireland by the Four Masters*, O'Donovan's translation; *The Annals of Clonmacnoise*, McGeogheahan's translation, a MS. in the library of Trinity College, Dublin. There are also *Grace's Annals of Ireland*, a MS. in the library of Trinity College, Dublin; and *The Book of Leinster*, a MS., McCory's translation, all quoted from the Census. Then he quotes A. D. 679-80, "a most grievous leprosy in Ireland, which is called bolgach;" in 1741, "leprosy in Ireland;" in 742-3, "the bolgach." The last entry is 1718-21, and in 1725 the practice of inoculation was introduced into Ireland (Q. 3,039-42).

(Q. 3,045.) In 1840 inoculation was prohibited by the 3d and 4th Vict., c. 29, entitled, "An act to extend the practice of vaccination." By this act payments were to be made to medical practitioners out of the public, or rather local public, funds for every vaccination performed. "The next act is a very important one." "The act of 1840 *enabled* guardians to do certain things, and I find that on reference to the records that the extent *to which they availed themselves of the powers given them almost steadily declined*." Then we come to the 14th and 15th Vict., c. 68, the Medical Charities Act, passed in 1851. It is to be noted that the guardians were *elected* officers, responsive, therefore, to some extent at any rate, to the wishes of the people; but by this act Ireland was divided into dispensary districts under *appointed* committees, who *appointed* medical officers, and they were to be paid for "free" vaccinations as before. Then came "the Vaccination (Ireland) Act 1858," further imposing vaccination on the people, and on 13th July, 1863, vaccination was made compulsory, under which act all children born after the 1st January, 1864, were to be vaccinated within six months of birth, and provides for payment to the vaccinators by the Board of Guardians (of course out of the public, union, funds).

In 1879 the time for vaccination was reduced to three months from birth. It increased the severity of the law, permitted (as construed by the courts) repeated convictions for neglect to vaccinate the same child. This Act tended to the more complete enforcement of the law for compulsory vaccination (Q. 3,046-7), and rendered the system much more effective. It enabled the *registrar*, an appointed officer, who is also generally the *vaccinating officer* interested pecuniarily in enforcing the rite, to institute proceedings to punish a recalcitrant parent instead of waiting for action by the Board of Guardians (elected officers). (Q. 3,049) Ireland is divided into 720 dispensary districts and 1,139 out-stations.

Dr. MacCabe proceeds to describe the machinery in its details for preventing escape from the meshes of the law, which it is not worth while to follow, except as illustrating the dangerous ingenuity evinced by persons upon whom special privileges are conferred (in this case a favored few of the medical profession) to secure the very last dollar they can gather in under force of the law.

Dr. MacCabe attaches great importance to the fact that when making their quarterly returns to the Local Government Board the medical officers are *required* to state whether they followed the instructions of the Local Government Board by seeking to produce four typical vesicles! and adds: "There is a good deal to be said upon that point presently."

It will be remembered that Dr. MacCabe was to tell all about the lymph used in Ireland, but it turns out that he knows nothing about it; nothing, that is, from a medical or scientific point of view. But of the commercial aspect he is well informed. He narrates the history of the Cow-pox Institution started by "three eminent physicians" in 1804, supported by subscriptions, by the sale of lymph, and by a subvention from the public funds of £400 ($2,000.00) per annum, increased in 1877 to £1,200 per annum, and then describes the number of "points" and Husband's tubes issued, and vaccinations performed (Q. 3,053-6). From the 14th January, 1804, to 15th June, 1889, 1,123,136 charged points and 28,516 Husband's tubes were issued, and 265,625 vaccinations were performed. Up to the 1st April, 1877, the points supplied to the poor Law Unions had been paid for by them, but in consideration of the increased government subsidy, the poor Law Unions were supplied gratuitously subsequent to that very appropriate date. Although no information is given as to the number supplied to the poor Law Unions, nor the amount received for the sale of the "points" and tubes, it is pretty evident that a very profitable business was here carried on.

At (Q. 3,059) he tells the Commission that he considers Ireland is *not* well protected in the way of vaccination. *His reason being that up to 1880 only two insertions were made.*

He also states that only four complaints have ever been made to the Local Government Board of any ill effects of vaccination since 1840. The first was in 1871; the second in the same year; the third in 1875; and the last in 1888; that "in each case an inquiry was held, and it was very clearly shown that the presumed injury inflicted by vaccination could not fairly be traced to the operation or to its effects."

At (Q. 3,062) he puts in "full particulars of the cases" (Appendix vii, p. 276, Second Report R. B. C.). The following is a copy of the "full particulars:"

"Summary of facts connected with complaints respecting the effects of (principally) Irish vaccine lymph supplies:

| Complainant. | No. | Union. | Summary of Facts. |
| --- | --- | --- | --- |
| Fras. Geogheegan. | 11,048/71 | North Dublin. | Complained of the fearful state his child was left in by vaccination. The medical officer (North City Dispensary) informed the Commissioners that he vaccinated eight other children with the same lymph as that with which Geogheegan's child was vaccinated, and that he inspected them afterward and found all of them in good health, and without any appearance of being similarly affected in the way complained of. |

Obs.: Dr. Willson, who vaccinated, states all the children of this family are liable to skin affections, and in one of these he had to postpone vaccination for three years on that account.

| Complainant. | No. | Union. | Summary of Facts. |
| --- | --- | --- | --- |
| Thos. Smyth. | 21,315/71 | Banbridge. | Complained that his child was vaccinated with impure lymph, and alleged that disease was thereby introduced into its system, from the effects of which it died. The Poor Law Commissioners obtained a report from the medical officer of the district (Tandragee), who denied that he used impure lymph, as he was very careful in selecting it, and also obtains supplies from the national vaccination establishment, and stated that if disease was introduced into the child's system it must have been subsequent to the operation. A copy of the medical officer's report was forwarded to the complainant, who thanked the commissioners for their attention in the matter, which was allowed to drop. |

Obs.: Twelve days after vaccination, the child, a twin, grew worse and worse, and lingered in intense pain for about nine days (twenty-one days after vaccination). Complainant (Mr. Smyth) believes "impure and diseased" lymph was used, the whole system became poisoned, and a tumor formed "in the side, from the effect of which it died." An assistant of Dr. Crawford performed the operation of vaccination in this case. This Dr. Crawford admits, and it is contrary to our regulations.

| Complainant. | No. | Union. | Summary of Facts. |
|---|---|---|---|
| Dr. Phelan (Graigne). | 2,565/75 | Thomastown. | Reported the case of a child alleged by him to have died from the effects of vaccination. The Local Government Board Inspector held an inquiry on oath into the circumstances of the case, and reported that after a careful consideration of the history of the child's life he was of opinion that vaccination was not either directly or indirectly the cause of the death of the child. |

Obs.: Child died from cerebral affection following dentition, attended with convulsions Dr. Phelan (the complainant) who was present at the inquiry, concurred in the opinion formed by the medical inspector who held the inquiry, that vaccination had not contributed in any way to the death; he withdraws his opinion and admits he formed a hasty conclusion on insuffícent evidence.

| Complainant. | No. | Union. | Summary of Facts. |
|---|---|---|---|
| Ellen Burke (Kilkee). | 12,876/88 | Vichush. | Complaint in reference to the death of her husband, on whom the operation of vaccination was performed by the porter of the dispensary. |

Obs.: Medical inspector was sent down to inquire into the whole of the facts, and the evidence taken (on oath) at the inquiry together with his report, and the certificate of death left no doubt that the man's death was due to well-marked *enteric fever*. He happened to have been revaccinated coincidently with the appearance of the first symptoms of his illness. The medical officer was severely censured for permitting the porter to vaccinate in this case.

If a person suspected of procuring the death of another by poison were to be selected to inquire into the cause of death, supposing he should think fit to admit that the suspected poison was actually found in the cadaver, he might be expected to report as in the above cases, that the poison in question had nothing to do with causing the death! The case is properly stated by Dr. Julius Hensel in his *Macrobiotic* (p. 119): "If a prosecuting attorney were to enter the Irish chamber with every disciple of Koch, Pasteur, and Jenner, and were to draw up a report in reference to the inoculation and its results, the friends of inoculation would be dissipated like mists by the sun. It is only their freedom from responsibility to common sense and the *criminal law* that causes the inoculators to imagine that they are in the right. They need to be brought back from this mistake by an accompanying *juge de paix*." Dr. MacCabe does not believe that any children are "insusceptible" (Q. 3,067). His experience is (Q. 3,068) that where insusceptibility is reported it indicates that the child has not been properly vaccinated. At (Q. 3,077) he is asked as to source of supply of lymph to the cow-pox institution, and how it is kept up. He answers the latter question, but not the former: "It is kept up by a number of collectors who are paid for the tubes they send in." The collectors are "medical men who have offered to charge the tubes."

(Q. 3,081-6.) The good doctor is taken by surprise at the Table F, App. 6, put in by Dr. Grimshaw, and partly prepared by himself. The number of "insusceptible cases" was so large as to give him "matter for very uncomfortable reflections."

At (Q. 3,080) Mr. Whithead asks: "You have given us some information from very early records in the history of Ireland as to the existence of small-pox at different times; is there any record of any recognized treatment of small-pox in vogue in those times?" "No, not that I am aware of. I have never heard it referred to."

(Q. 3,090.) "Nothing at all before inoculation?" "Nothing at all that I am aware of. *I believe historically that is the first record of anything which we have in the shape of*—it cannot be called curative treatment, but anticipatory treatment."

And yet, already by the year 1688 Sydenham's *Medical Observations Concerning the History and the Cure of Acute Diseases* had reached a third edition. (See Sydenham's works, published by the Sydenham Society.) In this work are three different dissertations: (1) On regular small-pox; (2) On the "anomalous small-pox of the years 1670-71-72," and (3) On the "irregular small-pox of the years 1674-75." In the first, while treatment is spoken of throughout the dissertation, pp. 138-151 are devoted exclusively to it. In the second, two pages are so given, and in the third, four pages. The treatment recommended by Sydenham will bear comparison with that given in the very newest and most standard works on the practice of medicine of the dominant school, to which it is pretty certain Dr. MacCabe and all the other witnesses *for* vaccination belong. It is also to be borne in mind that it was the long and successful use of his treatment which led to Sydenham's declaration in 1688 of the little seriousness of small-pox unless improperly treated.

Mr. Picton tried (Q. 3,096) to get from Dr. MacCabe the source of the "lymph" employed, and is told that the vaccinators always report that they use that supplied from the local government establishment (see above, Q. 3077), or that they keep up supplies from arm to arm.

(Q. 3,097.) Some animal lymph has been used; Dr. Montgomery procured some from Dr. Warlomont and had good results, but at (Q. 3,115) Dr. MacCabe corrects this statement; Dr. Montgomery gets it from a Mr. Darke, of London, and *perhaps he gets it from* Dr. Warlomont.

(Q. 3,127-8.) Mr. Darke has no public position.

(Q. 3,130.) "You simply send for this lymph and take it on faith?" "So it would appear."

(Q. 3,098.) Mr. Picton questions Dr. MacCabe about the "marks." One mark will afford considerable protection for a time, and though the vaccinee runs great risk of contracting small-pox when adult, he certainly will not have as bad an attack as if he did not bear any mark.

On what data can Dr. MacCabe or any one else found this assertion? How does he know; how can any one know how seriously Nature is going to visit any one with small-pox? When small-pox kills, can it be said that the attack is less bad than it would have been had the victim not been vaccinated? Was Dr. MacCabe ignorant that tens of thousands of the vaccinated have died of small-pox? Statistics give no warrant for his statement, for they show that the fatality of small-pox to-day (all cases included) is certainly not less than it was in pre-vaccination days.

The assertion is but an illustration of the entire fabric of bold, baseless assertion on which this "grotesque superstition" (see *post*, testimony of Dr. Creighton) has been built and maintained from Jenner down to this day.

But (Q. 3,099) he considers that four marks will "continue the protection." He has never met with a single case of a person taken with small-pox who showed four marks.

In 1879 the Guardians of the Galway Union proposed to inoculate a calf with small-pox, so as to obtain a full supply of lymph for the Union (Q. 3,104-7), but by letter 10th February, 1879, the Local Government Board for Ireland objected to this being done, stating that if the inoculation were to be with small-pox virus small-pox would be propagated, and the operator would be liable to prosecution under the Act 31st and 32d Vict., cap. 87, forbidding small-pox inoculation; the letter also went on: "If the proposition were to vaccinate a calf with lymph obtained from a human subject the Board have to state that it has *long since* been *ascertained* that animal lymph for vaccination purposes must, in the first instance, be obtained from a cow, in which the disease has

*spontaneously* arisen, and that vaccination performed with lymph taken from a cow which had been vaccinated with human lymph is not reliable."

But in answer to (Q. 3,108-9) Dr. MacCabe expresses his dissent from the views expressed in that letter, and says: "Of course if a calf were inoculated with the virus of small-pox the resulting lymph, if any result followed, for it is a very difficult thing to do I believe, but if any result followed the *lymph taken from the calf would be cow lymph*, and would not communicate small-pox. * * * If a calf had been vaccinated with humanized lymph I suppose everybody who has studied the subject would say that no result at all would follow."

(Q. 3,110.) "Should I correctly understand your opinion to be that while it is difficult to inoculate small-pox upon a cow or calf, the lymph which could be obtained from such a source would be cow-pox and not small-pox?" "That is my opinion."

(Q. 3,110.) "You would regard the vaccine disease as small-pox grafted on the cow?" "Quite so."

(Q. 3,112.) With reference to spontaneous cow-pox he is not an expert. "All that I know in connection with calf lymph is solely from reading, and the authority that has guided me I think is Dr. Seaton principally."

(Q. 3,113.) "As one evidently acquainted with the literature of the subject perhaps you are aware that Dr. Edward Jenner considered spontaneous cow-pox to be worthless as a protection?" "I canot say that I remember that, I do not doubt that it is so."

(Q. 3,114.) On p. 7 of his *Inquiry* he says: "It is necessary to observe that pustulous sores frequently appear spontaneously on the nipples of the cows, and instances have occurred, though very rarely, of the hands of the servants employed in milking being affected with sores in consequence, and even of their feeling an indisposition from absorption. These pustules are of a much milder nature than those which arise from that contagion which constitutes the true cow-pox. They are always free from the bluish or livid tint so conspicuous in the pustules in that disease. No erysipelas attends them, nor do they show any phagedenic disposition as in the other case, but quickly terminate in a scale without creating any apparent disorder in the cow. This complaint appears at various seasons of the year, but most commonly in the spring when the cows are first taken from their winter food and fed with grass. It is very apt to appear also when they are suckling their young. But this disease is not to be considered as similar in any respect to that of which I am treating, as it is incapable of producing any *specific effects on the human constitution.* However, it is of the greatest consequence to point it out here lest the want of discrimination should occasion an idea of security from infection of the small-pox which might prove delusive"? "I remember reading that perfectly well, many years ago."

(Q. 3,132.) He believes there are a variety of eruptions upon the teats of the cow, but (Q. 3,133-7) he has never seen them; he is not an expert; he knows nothing upon the subject except from his reading; he knows no one in Ireland who can be referred to as an expert, but he "should imagine the officers of the Local Government Board here" are such experts.

(Q. 3,140.) "Do you think any of the post-vaccinal small-pox that occurs has reference to the use of spurious cow-pox lymph?" "I am not prepared to answer that question."

(Q. 3,141.) By Mr. Picton: "Could you explain to an unprofessional man what vaccination is?" "I think I had better not attempt to do it."

(Q. 3,142.) "You judge only by results?" "Yes."

(Q. 3,143.) "That is, what prevents small-pox is vaccination, and what does not prevent it is not proper vaccination?" "Improper vaccination is not vaccination at all, but I have never seen improper vaccination in Ireland. I have never yet seen a vesicle produced by vaccination in Ireland that was not a typical vaccine vesicle."

Then according to his statement (Q. 3,098 above) every person vaccinated in Ireland, *though only with one mark*, is protected at least to the extent that his cannot be so bad a case as if he had not been vaccinated; there cannot be a worse than a fatal case; therefore no such person ought to die of small-pox in Ireland. But Dr. Grimshaw's table K (p. 274, App. No. 6, 2c Rep.) shows not less than seventy-four persons whom he admits were vaccinated *did* die of small-pox during the eight years 1881-88; of these, eight were under five years of age, eleven under ten, and eighteen under fifteen years of age.

(Q. 3,144-6.) By Dr. Savory: "You would recognize something characteristic which follows the insertion of the lymph?" "Yes." "Which you can diagnose or distinguish?" "Yes." "There can be no question about that?" "No."

(Q. 3,147.) That which follows the insertion of the lymph is so characteristic that it is impossible for a practical vaccinator to be mistaken.

(Q. 3,149.) By Dr. Collins: "Are you equally satisfied that there is no other pox upon the teats of cattle that will produce the same phenomena?" "I do not know that there is."

(Q. 3,150.) "I should know a true Jennerian vesicle, and I think it would be impossible for me not to be able to distinguish between that and spurious pox, not only upon its actual appearance from the date of vaccination to the falling off of the scab, but also in the typical and unmistakable appearance of the resulting cicatrix—it is impossible to mistake it for anything else."

(Q. 3,151.) "You are not acquainted with any other virus that would produce the same phenomena?" "No."

